# Research on the Impact of Green Innovation Network Embeddedness on Corporate Environmental Responsibility

**DOI:** 10.3390/ijerph20043433

**Published:** 2023-02-15

**Authors:** Junli Wang, Wendong Lv

**Affiliations:** Business School, University of International Business and Economics, Beijing 100029, China

**Keywords:** innovation network embeddedness, corporate environmental responsibility, green reputation

## Abstract

In the process of China’s economic transformation, enterprises urgently need to use green innovation networks to realize corporate sustainability. Based on resource-based theory, this study explores the internal mechanism and boundary conditions of green innovation network embeddedness that affect corporate environmental responsibility. This paper conducts an empirical study based on panel data of listed companies engaged in green innovation in China from 2010 to 2020. Drawing on network embeddedness theory and resource-based theory, we found that relational and structural embeddedness influenced green reputation, which affected corporate environmental responsibility. We also identified the importance of ethical leadership and examined its role in moderating the effect of green innovation network embeddedness. A further investigation revealed that the impact of network embeddedness on corporate environmental responsibility was particularly pronounced in the samples of enterprises with high-level political ties, loose financing restrictions, and nonstate ownership. Our findings highlight the advantages of embedded green innovation networks and offer theoretical references and recommendations for enterprises considering network participation. Enterprises should attach great importance to the network embedding strategy of green innovation for corporate environmental responsibility and actively integrate the concept of green development into network relationship embedding and network structure embedding. Moreover, the relevant government department should provide necessary environment incentive policies according to the enterprise’s development needs, especially for the enterprises with low-level political ties, high financing restrictions, and state ownership.

## 1. Introduction

Corporate environmental responsibility is integral to corporate social responsibility, closely related to corporate sustainability [1]. Especially with the rapid growth of China’s economy, its environmental problems are becoming increasingly prominent. The scholarly community has turned its attention toward figuring out how to fulfill the objective of sustainable growth of man and nature. Enterprises play a crucial role in nations’ economic, social, and technological development as significant contributors to economic activity, significant employers, and significant promoters of technological advancement, which is a major force for environmental responsibility [2]. Recently, corporate environmental responsibility has changed from a simple “business responsibility” to a strategic competitive resource. The performance of corporate environmental responsibility will be used as the basis for purchasing or selling when institutional investors in the industry, who have access to increasing amounts of capital, make investment decisions. However, what has plagued companies as they take on environmental responsibilities is balancing their particular interests and broader social goals. At present, it is widely recognized by the government, investors, consumers, and other stakeholders that green innovation activities can help enterprises balance economic and social benefits [3]. An increasing number of businesses are incorporating the concept of corporate social responsibility with “green innovation” consciousness. Green innovation activities involve many participants, such as governments, service providers, banks, scientific research institutions, and supply chain enterprises [4]. How to coordinate the relationship between all parties and conduct practical cooperation reflects the complexity of the green innovation process [5,6,7]. In other words, the more frequent network activities among enterprises, the more likely they are to achieve green innovation and benefit from it [8,9,10]. Some studies have shown that by incorporating such a vast open system as the green innovation network, all innovative subjects in the network collaborate, promote the vitality and potential of green innovation within firms, and assist enterprises in reducing environmental pollution [3,11], which improves resource and energy utilization efficiency [12,13], reduces ecological degradation, and increases social welfare [14]. In addition, these enterprises also establish an excellent green reputation and increase stakeholder trust in the enterprise [12,15], which enhances the enterprise’s commitment to corporate environmental responsibility. Thus, green innovation network embeddedness plays a vital role in improving their corporate environmental responsibility performance. It has become crucial to thoroughly investigate how Chinese businesses might increase their corporate environmental responsibility through the embeddedness of green innovation networks.

First of all, the existing research generally believes that innovation network embeddedness will significantly impact enterprise economic performance. For example, some researchers suggest that innovation network embeddedness can promote the enterprises’ financial performance (such as their net profit margin, return on equity, and return on assets) [16,17] and innovation performance (such as the enterprises’ new product development, innovation efficiency, patent number, and other indicators) [18,19]. Additionally, although the study has shown that networks could affect corporate social responsibility [20], the way that green innovation networks’ embeddedness affects the corporate environment remains to be studied. Additionally, the methods used in the extant literature on network embeddedness are still theoretical analyses and questionnaire surveys. Extensive sample empirical analysis is rarely used in research to give this kind of proof. Therefore, this paper first intends to explore the impact of green network embeddedness on corporate green environmental responsibility based on the data of listed companies, which is the first research question.

Secondly, most current research on network embeddedness follows the traditional theoretical logic of “network embeddedness-resources/capabilities-enterprise performance”. However, few studies take green reputation as the mediating variable to discuss the impact of network embeddedness on corporate environmental responsibility. The corporate social network is an informal mechanism for enterprises to obtain resources. Networks’ embeddedness can provide valuable, rare, hard-to-imitate, and hard-to-replace resources. These resources have the potential to boost businesses’ profits and give them a competitive edge [21,22,23]. In the era of the green economy, a green reputation is regarded as a valuable and intangible asset that cannot be duplicated. The green innovation network embeddedness will influence enterprises’ green economic operations, which will have a variable impact on the green reputation by stakeholders [24,25]. In addition, Friedman and Miles [26] proposed that corporate reputation may be one of the main drivers of corporate social responsibility. A good reputation will affect the long-term viability of enterprise value, organizational performance, and financial status [27,28,29]. However, few studies have incorporated green reputation into green innovation network resources to explore how network embeddedness can enhance corporate environmental responsibility by obtaining a green reputation.

Finally, green innovation network embeddedness has a situational dependence on the mechanism of corporate environmental responsibility. However, few studies have explored the moderating effect of leader characteristics on network embeddedness. Therefore, the third research question explores the boundary conditions between the embeddedness of green innovation networks and corporate environmental responsibility. However, previous literature has explored the moderating effect of network embeddedness. For example, internal factors include absorptive capacity and internal value creation capacity [30,31], and external factors include environmental dynamics and network models [32,33]. However, there is still a lack of further research based on leader characteristics. Undertaking corporate environmental responsibility requires enterprises to pay a specific cost. Leaders’ ethics can complement environmental regulation when it is challenging to implement it. As Fu et al. [34] believed, in the sustainable development strategy, the individual differences of leaders can affect the extent to which enterprises make beneficial activities.

Based on this, we aim to (1) reveal the impact of green innovation network embeddedness on corporate environmental responsibility; (2) reveal the mediating effect of green reputation between green innovation network embeddedness and corporate environmental responsibility based on resource-based theory; (3) Explore the boundary mechanism of ethical leadership in the logic chain of “green innovation network embeddedness, green reputation, corporate environmental responsibility”.

In the CPC patent classification system issued by the European Patent Office and the United States Patent Office, this paper takes Y02 and Y04 classification as essential indicators of green patents and builds a green innovation network based on the green patents issued by Chinese A-share listed companies from 2010 to 2020. Moreover, this paper introduces Chinese enterprises’ green innovation network embeddedness through a large sample empirical study and explains whether the green innovation network embeddedness improves corporate environmental responsibility. It was found that both relational embeddedness and structural embeddedness can significantly promote corporate environmental responsibility. In terms of a mediating mechanism, green innovation network embeddedness aids in enhancing enterprises’ green reputation, and green reputation supports firms’ commitment to corporate environmental responsibility. Further analysis shows that ethical leadership positively regulates the intermediary effect of green reputation between green innovation network embeddedness and corporate environmental responsibility. In addition, embeddedness in green innovation networks in fostering corporate environmental responsibility is especially apparent in enterprises with high-level political ties, no financial constraints, and nonstate ownership. A variety of robustness and endogenous testing strategies are used to improve the unbiased and consistent estimation of the model.

We intend to make the following contributions: (1) By integrating “green innovation” and “network embeddedness”, we explore the social consequences of green innovation network embeddedness and expand the application of network embeddedness theory in the field of green innovation. (2) This study expands on previous research on the relationship between green innovation network embeddedness and corporate social performance. It explores the implications of green innovation network embeddedness on corporate environmental responsibility. (3) It provides a theoretical foundation for enterprises to play the beneficial function of network embeddedness in corporate environmental responsibility. It increases their awareness of the driving forces behind corporate environmental responsibility. (4) By introducing resource-based theory, this study examines the intermediary role of “green reputation” between green innovation network embeddedness and corporate environmental responsibility. Furthermore, it reveals the impact of network embeddedness on green resources and their economic consequences. (5) The scope of existing network embeddedness theory research is increased by looking at the moderating role of ethical leadership in the green innovation network embeddedness affecting corporate environmental responsibility, and the theoretical development of the green innovation network embeddedness process affecting corporate performance is enhanced.

The rest of this paper is organized as follows: The second part is the theoretical grounding and hypothesis development. The third part is the research design. The fourth part is the empirical test results and analysis of the research hypothesis. Finally, we summarize the theoretical contributions, put forward the corresponding practical suggestions, and point out the shortcomings of the research and future research directions.

## 2. Theoretical Grounding and Hypothesis Development

### 2.1. Green Innovation Network Embeddedness and Corporate Environmental Responsibility

Granovetter [35] first classified network embeddedness into relational and structural embeddedness, the most widely used classification in network embeddedness research. “Relational embeddedness” means that the behavior subject is embedded in the relationship network in which it is located, and its behavior is affected by its social relationship network, emphasizing the relationship characteristics between the enterprise and other members of the network. Relational embeddedness is a close and special cooperative relationship that mainly focuses on the mutual relationship under the trust mechanism. The strong tie is the primary indicator of “relational embeddedness” [36]. In contrast to “relational embeddedness”, “structural embeddedness” refers to the position of node enterprises within a sizeable social relationship network and encompasses a broader range of actors. The centrality metric is used in this paper to represent the “structural embeddedness”. A higher degree of network centrality gives enterprises more network power, making it easier to obtain and control new information related to innovation in the network [37].

Relational embeddedness has two critical effects on corporate environmental responsibility. Maintaining a close network connection between enterprises can give them flexibility and speed, so they can change their plans in response to shifting market conditions [38,39]. Therefore, information exchange through solid ties is essential for enterprises to fully understand the social demand for green technology. Taking customers’ preference for environmental protection products as an example, this external information helps enterprises better meet these needs in the process of green innovation to assume their environmental and social responsibilities better. For another, the basis of interest in social networks is the value generated by mutual trust between the two sides [40], particularly the connection state of frequent interaction, close emotion, close ties, and mutual benefit exhibited by strong relationships [35], which can offer productive value [41]. This cooperative relationship has a solid exclusive feature: the resource demands between the two parties are relatively single minded. In this situation, enterprises may further solidify mutual trust and build the groundwork for long-term cooperation if they actively uphold their social obligations and defend the rights and interests of stakeholders [42]. Therefore, in the green innovation network, enterprises with solid ties are often conducive to promoting enterprises to fulfill their environmental and social responsibilities.

Regarding the effect of structural embeddedness on corporate environmental responsibility, it is essential to note that by taking on environmental and social duties, central enterprises can improve their competitive advantages in green resources. This is because the higher the centrality of an enterprise, the more information and resource channels it can access and the more potent its ability to access and control resources [21,33]. However, it is not always possible to fully manage the timeliness and accuracy of the data and resources that significant firms can access. Instead, it mostly depends on the willingness of marginal enterprises [43]. Therefore, to obtain more critical environmental information and resources from the green innovation network, central enterprises need to be supported by marginal enterprises to establish a long-term cooperation model. A lack of legitimacy and trust will reduce the willingness of edge enterprises to support information and resources [44]. Enterprises can achieve consistency in their own economic and social goals and legalize their economic operation by actively taking on environmental and social responsibilities. They can also gain the trust of marginal enterprises by doing this, which is beneficial for forming long-term cooperative relationships and lowering risks [45,46,47].

On the contrary, it is simple to be exposed to dire repercussions or even be imitated if the firm does not actively meet its environmental and social duties. This concern enables stakeholders to have governance effects on enterprises with high centrality. Based on this, enterprises usually respond positively to pressure and supervision from stakeholders [48]. Therefore, enterprises in strategic locations are more likely to be prepared to take on corporate environmental responsibility and have higher green ratings, strengthening their advantages in accessing green resources. To sum up, we propose the following:

**Hypothesis** **1a.** *Relational embeddedness is positively related to corporate environmental responsibility.*

**Hypothesis** **1b.** *Structural embeddedness is positively related to corporate environmental responsibility.*

### 2.2. Network Embeddedness, Green Reputation, and Corporate Environmental Responsibility

The impact of green innovation network embeddedness on green reputation mainly includes three points. Granovetter [35] believed that the so-called “embeddedness” means that economic activities will be limited by the social structure and social relations in which they are located, which determine the form and results of economic activities. “Embeddedness” is a process in which social structure and relations affect economic activities. That is, the embeddedness of green innovation networks will affect the green economic activities of enterprises. However, a comprehensive assessment of an enterprise’s green reputation is made when stakeholders take into account its historical environmental protection practices and future potential. That is, the past green economic behavior of enterprises will inevitably lead to stakeholders’ evaluation of corporate reputation [24,25]. Therefore, the embeddedness of the green innovation network will influence enterprises’ green economic operations, which will have a variable impact on stakeholders when assessing a green reputation. That is, green innovation network embeddedness will affect the green reputation of enterprises.

1. Relational embeddedness and Green Reputation

First of all, if network members can forge close bonds of cooperation, they place a higher value and regard on one another [49]. That is, if two individuals are closely connected, it will affect their positive evaluation of each other [50]. Strong ties indicate that network members have high levels of trust, frequent interactions, closeness, and reciprocity [51]. This also implies that network members have rich emotional connections, which will strengthen the positive evaluation between the two sides and subsequently positively affect corporate reputation. Furthermore, reputation is the result of signal diffusion [52]. Through embedding green innovation network embeddedness, enterprises can show positive green behavior in their communication and interaction with other network members, thus releasing positive signals. Therefore, the more supportive the network relationship is of its green conduct, the more supportive it is of developing a superb green image among stakeholders, and the more supportive it is of increasing the green reputation of enterprises.

Secondly, information asymmetry is the main reason for opportunistic behavior. However, strong relationships entail a high level of trust between businesses and ongoing collaboration and contact. The degree of information asymmetry between the two parties is significantly reduced, and the higher the cost of enterprises breaking the network contract or engaging in opportunity-costing behavior [33,53,54]. Therefore, with the deepening of cooperation, enterprises will pay more attention to managing their own environmentally friendly conduct, signal that they are actively doing so, and enhance their environmental reputation.

Finally, effective green management must be the foundation for developing a green reputation. In the green innovation network, partners with strong ties often communicate frequently, which helps enterprises learn rich green management knowledge, including establishing good green management awareness, establishing green management organizations, improving green reputation management systems, cultivating green reputation management talents, and focusing on green reputation management communication. Therefore, strong ties can influence corporate green reputation by influencing enterprises’ learning of green management knowledge.

2. Structural embeddedness and Green Reputation

First, according to resource-based theory, the ability of an enterprise to create value depends on the resources it has. Corporate reputation is a comprehensive evaluation of the past behavior of enterprises by stakeholders, which reflects the ability of enterprises to provide valuable output to stakeholders [52]. Enterprises in a central location can connect with other users in the network and widen the channels for resource acquisition thanks to the benefits of their network placement [21]. Additionally, centrality shows the network’s control over and reliance on the green resource. Due to their advantageous location, more external organizations may request information from them, and central enterprises’ perceived impact will also grow [41]. Green information and resources are shared among enterprises and can spread in the network, thus changing the reputation evaluation of enterprises by stakeholders in the network [55]. Therefore, this ability to access and control resources by using the network’s central location will inevitably affect stakeholders’ evaluation of the enterprise’s green reputation [56,57].

Secondly, maintaining tight ties with trustworthy businesses can help them build their reputation because a good reputation can have ripple effects. This is especially true when partners have a robust technological base and a competitive advantage [58]. While obtaining diversified resources, the central enterprises can contact diversified partners, allowing them to cooperate with enterprises with high green reputations. Therefore, through the careful selection of partners, the businesses in the center decide to work with high-quality businesses with a solid reputation for being environmentally friendly and a wealth of green resources [59]. This helps them build their good reputations for being environmentally friendly.

Finally, many studies have shown that network embeddedness prevents companies from simply engaging in opportunistic activity since the repercussions are severe and others can quickly discover their behavior [54]. The network’s central location is a symbolic location, which has a significant symbolic effect. Once the reputation is damaged, the loss will be more significant [60]. Therefore, enterprises in the central position will cherish their reputation more, restrain their behavior, and release good green behavior signals. In addition, a network system’s reputation is crucial for regulating and constraining behavior. A good reputation is crucial for choosing whom to collaborate with and whom to avoid, further enhancing reputation [55].

3. Green reputation and corporate environmental responsibility

According to stakeholders’ references to particular standards, enterprise green reputation is a comprehensive assessment of the past green environmental protection behavior and future possibilities of firms [47]. In the era of a green economy, a green reputation is regarded as a valuable and intangible asset that cannot be imitated and an important symbol of the soft power of enterprises [61]. First of all, social responsibility requires a specific cost. An excellent green reputation can send a positive signal, which is an essential basis for financial institutions to provide loans. Therefore, the better the green reputation, the easier it is for enterprises to obtain credit financing and show good performance levels [62]. Therefore, enterprises with an excellent green reputation are more capable of assuming corporate environmental responsibility.

Secondly, a green reputation can significantly enhance investors’ confidence in the future profitability of enterprises. Previous studies have shown that institutions with high reputations may have greater legitimacy, thus enhancing their ability to attract funds, customers, information, and other resources [55,63]. Therefore, enterprises with high green reputations tend to show higher expected stock returns [64]. An excellent green reputation is believed to enable the company to obtain a premium and attract investors in the capital market more quickly. Because of this, enterprises with strong green reputations will be more driven to uphold their environmental and social obligations and send out positive environmental signals by disclosing information about their corporate environmental responsibility, which is a crucial foundation for investors to use when making investing decisions. Finally, the characteristics of a green reputation are long term and vulnerable [65]. Long term refers to cultivating and accumulating a green reputation, which requires a lot of human, financial, and material resources. It is a long-term accumulation process. Vulnerability means losing a green reputation is a short-term and fragile process. Once an adverse event occurs, the long-term accumulated reputation of an enterprise may be destroyed. In order to protect their green reputation from being disparaged at will, avoid ethical issues, and gain the competitive advantage provided by corporate environmental responsibility, enterprises will pay more attention to controlling their behavior and actively fulfilling their environmental and social responsibilities as their green reputation continues to grow. Promoting a green reputation will therefore encourage enterprises to fulfill their social obligations actively.

To sum up, we propose the following:

**Hypothesis** **2a.** *Relational embeddedness indirectly affects corporate environmental responsibility via green reputation.*

**Hypothesis** **2b.** *Structural embeddedness indirectly affects corporate environmental responsibility via green reputation.*

### 2.3. The Moderating Effect of Ethical Leadership

First of all, by encouraging internal employees to imitate and learn from the leaders’ “ethical models” so that more employees agree with the necessity of green production, ethical leaders can more accurately identify the needs and expectations of the social system on environmental norms and raise the enterprise’s overall environmental awareness [66]. At the same time, ethical leaders are more inclined to care for their subordinates and create a leadership image of integrity, honesty, and clear rewards and punishments [67]. This leadership image helps form a resource-saving corporate culture, promotes employee communication and knowledge sharing, and improves resource-use efficiency [68]. A solid green culture can motivate businesses to innovate more sustainably through network connections, to operate sustainably by environmental protection standards, to promote green products more quickly, and to develop positive environmental perceptions of their brands. Secondly, ethical leaders can use their right to coordinate essential resources, provide the resources needed by employees, develop their green creativity, take into account staff suggestions for green innovation [69], and master the ability to build and maintain relationships with various stakeholders during business operations.

When making business decisions, the ecological environment will be considered by creating an incentive system to promote cooperation and strengthen the commitment and motivation of sustainable development [70], such as offering customers green products and services. Therefore, as enterprises are deeply embedded in the green innovation network, stakeholders will perceive the importance that enterprises attach to environmental protection to enhance the evaluation of enterprises’ green reputation. Finally, ethical leaders tend to care for their subordinates by creating a leadership image of integrity, fairness, and clear rules of reward and punishment in their employees’ minds [67]. Therefore, ethical leaders are likelier to obtain “trust rewards” from employees. Employees will thus feel more compelled to demonstrate behaviors at work that are advantageous to the company and endeavor to raise their level of performance, which will aid the company in exhibiting commendable green behavior and enhancing its green reputation. To sum up, ethical leadership can enhance the positive effect of green innovation network embeddedness on corporate green reputation and ultimately enhance corporate environmental responsibility. To sum up, we propose the following:

**Hypothesis** **3a.** *Ethical leadership positively moderates the effect of relational embeddedness on green reputation.*

**Hypothesis** **3b.** *Ethical leadership positively moderates the effect of structural embeddedness on green reputation.*

**Figure 1:**
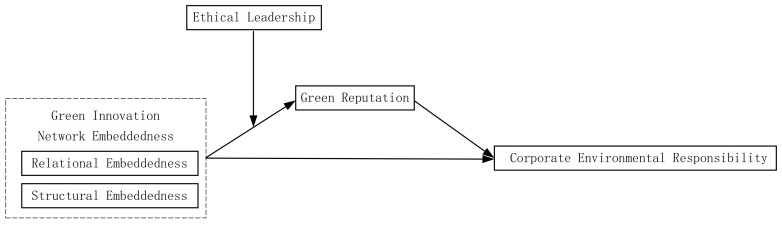
Hypothesized research mode.

The conceptual framework of this study is shown in Figure 1:

## 3. Research Design

### 3.1. Sample Selection and Data Collection

This study takes Chinese A-share listed enterprises engaged in green innovation as an example to conduct empirical research. The data were downloaded from the IncoPat patent database and the WIND database of Clarification Analytics. The specific data processing steps are listed: First, search the IncoPat commercial patent database for all the green patent data applied by each A-share listed enterprise from 2010 to 2020. The second step is to use Python to split the applicant’s obtained patent data and match the enterprise’s listed stock code to facilitate the subsequent indicator matching and fusion processing. The third step is data cleaning, excluding enterprises that do not match the stock code and those that do not submit patent applications during the observation period. Finally, we obtained a total of 534 Chinese A-share listed enterprises that have conducted green innovation from 2010 to 2021. The fourth step is index calculation, which uses pandas, networks, and other packages in Python to build interconnected networks or models. The year and the five years serve as the foundation for calculating indicators. The annual solid ties and centrality of businesses in the corporate green innovation network are calculated using each year as the time unit in the robustness test.

In order to obtain more reliable data, we followed the following steps: (1) exclude ST and PT company samples; (2) remove the company samples with missing data values of relevant indicators; (3) remove the company samples with abnormal data values of relevant indicators; (4) exclude the sample of financial industry companies; (5) winsorize continuous variables.

### 3.2. Model Design

We designed the following models to test the hypothesis of this paper.

First, in order to consider the impact of network embeddedness on corporate environmental responsibility (Hypothesis 1a and 1b), we designed the following models:

CER_i,t_ = β_0_ + β_1_NR_i,t_ + γControls + γYear + γIndustry + ε_i,t_ (1)

CER_i,t_ = β_0_ + β_1_NS_i,t_ + γControls + γYear + γIndustry + ε_i,t_ (2)

Among them, CER is the proxy variable of corporate environmental responsibility. NR is the proxy variable of relational embeddedness, while NS is the proxy variable of structural embeddedness. The control variables include firm age (Age), capital structure (Lev), operating profit ratio (Profit), enterprise growth (Growth), corporate ownership (Soe), corporate value (CV), cash holdings (Cash), board size (Board) and proportion of independent directors (Indep), i and t represent enterprises and years, respectively, and ε_i,t_ represents the residual items.

Secondly, in order to discuss whether green reputation plays a mediating effect (Hypothesis 2a and 2b), we used a three-step method and designed the following models:

CER_i,t_ = β_0_ + β_1_NR_i,t_ + γControls + γYear + γIndustry + ε_i,t_ (3)

GR_i,t_ = β_0_ + β_1_NR_i,t_ + γControls + γYear + γIndustry + ε_i,t_ (4)

CER_i,t_ = β_0_ + β_1_NR_i,t_ + β_2_GR_i,t_ + γControls + γYear + γIndustry + ε_i,t_ (5)

CER_i,t_ = β_0_ + β_1_NS_i,t_ + γControls + γYear + γIndustry + ε_i,t_ (6)

GR_i,t_ = β_0_ + β_1_NS_i,t_ + γControls + γYear + γIndustry + ε_i,t_ (7)

CER_i,t_ = β_0_ + β_1_NS_i,t_ + β_2_GR_i,t_ + γControls + γYear + γIndustry + ε_i,t_ (8)

Among them, the mediating variable GR is the proxy variable of green reputation, and other variables are the same as above. Suppose the β_1_ in the model (3) and model (6), β_1_ in the model (4) and model (7), and β_2_ in the model (5) and model (8) are significant. In that case, the mediating effect—the function of green reputation in mediating the relationship between network embeddedness and corporate environmental responsibility—is also significant.

Finally, in order to check whether ethical leadership has a moderating effect on the relationship between green innovation network embeddedness and green reputation (Hypothesis 3a and 3b), we designed the following models:

CER_i,t_ = β_0_ + β_1_NS_i,t_ + β_2_NR*Eth_i,t_ + γControls + γYear + γIndustry + ε_i,t_ (9)

CER_i,t_ = β_0_ + β1NR_i,t_ + β2NS*Eth_i,t_ + γControls + γYear +γIndustry + ε_i,t_ (10)

Among them, the moderating variable Eth is the proxy variable of ethical leadership, and the other variables are the same as above. If the coefficient of the interaction term (β_2_) in model (9) and model (10) is significantly positive, Hypotheses 3a and 3b are supported. This means that Ethical leadership positively moderates the effect of network embeddedness on green reputation.

### 3.3. Measurements

1. Corporate environmental responsibility

CSR is a kind of international private business self-regulation [71]. With increasingly serious environmental problems, environmental protection has become an essential part of corporate social responsibility. Corporate environmental responsibility mainly refers to the responsibility of enterprises for environmental pollution control and ecological environment protection [2,72]. This study chose the environmental responsibility ratings in the social responsibility report evaluation method of Hexun-listed firms to measure corporate environmental responsibility. The original data came from the social responsibility reports and annual reports released by the listed companies of the Shanghai Stock Exchange and the Shenzhen Stock Exchange through official websites.

2. Network embeddedness

In the green innovation network, relational embeddedness (NR), typically assessed by solid ties, primarily assesses the level of trust, time investment, emotional engagement, and reciprocity among related businesses. Referring to the quantitative analysis method of Phelps [73], we used the total number of times enterprises and their partners participated in green patent cooperation to measure the strong tie.

Structural embeddedness (NS) refers to the impact of the relative position of enterprises in the green innovation network on enterprises. We used centrality as a measurement indicator. The higher the centrality, the more enterprises are in the core position of the network. The commonly used indicators of centrality include degree centrality, intermediate centrality, and proximity centrality. Any of the three can be chosen because the calculation results are close. This paper selected the commonly used degree of centrality to measure network centrality. The calculation formula is C (n_i_) = d (n_i_)/n − 1, where n is the total number of nodes, d(n_i_) = ΣjX_ij_ when the node n_i_ is not adjacent to n_j_, and X_ij_ = 0 when node n_i_ is not adjacent to n_j_, X_ij_ = 1.

3. Green reputation

We describe corporate green reputation as an overall assessment of past green environmental conduct and firms’ future prospects based on stakeholders’ reference to particular standards based on Fombrun’s [52] definition of corporate reputation. Fortune first conducted the ranking of corporate reputation in the United States. It was scored by external directors, financial analysts, and senior managers based on eight perspectives. This survey method has been adopted in many academic studies. This paper uses the environmental management system certification score to measure green reputation. Generally speaking, the better the green reputation is, the higher the environmental management system certification score will be.

4. Ethical leadership

The concept of “ethical leadership” was created due to ethics’ increasing value in both industry and academics. Ethics is seen as a crucial component of a leader’s qualities. According to Brown et al. [74], ethical leadership is a leadership style that models normative and preferable behaviors through personal acts and interpersonal interactions and encourages similar behaviors in followers through two-way communication, positive reinforcement, and decision making. We measured ethical leadership from two aspects, including the humanistic care orientation and ecologically sustainable development orientation, drawing on research from Jones et al. [75], Brown et al. [74], and Wang et al. [76]. The total score of ethical leadership was obtained by summing up the scores of all indicators of the two dimensions.

5. Control variablesp

Regarding the related research [77,78,79], this paper controls three variables that affect corporate environmental responsibility. (1) The first category is the essential characteristics of the company, including the firm age, capital structure, cash holdings, and company value. In fulfilling their social responsibilities, older enterprises attract more attention from the public and often shoulder more social responsibilities. At the same time, CSR will waste capital and other resources and put the company at a competitive disadvantage compared to companies that undertake more CSR activities, thus reducing the value of the enterprise [80]. This paper obtained the age data of enterprises by calculating the length of time from the establishment date to the observation period (Table 1). The asset–liability ratio measures the capital structure. Cash holdings were calculated as the difference between the book value of all assets minus the short-term investments of monetary funds and the sum of cash and short-term investments. The company value was measured by the ratio of the market value of the owner’s equity and liabilities to the company’s total assets. (2) The second category is company performance characteristics, including the operating profit ratio and enterprise growth. The financial performance of an enterprise may affect its investment in research and development, employee compensation and welfare, environmental protection, etc., thus affecting the fulfillment of environmental responsibilities. Enterprise growth is determined by the operating revenue growth rate year over year, and the operating profit determines the operating profit ratio to the total operating revenue ratio. (3) The third type is corporate governance characteristics, including ownership, the proportion of independent directors, and board size. CSR is an extension of firms’ efforts to foster effective corporate governance, ensuring sustainability via sound business practices that promote accountability and transparency [81]. These three variables can control different levels of corporate governance. Specifically, “1” refers to a state-owned enterprise, and “0” refers to a non-state-owned enterprise. The proportion of independent directors is defined as the ratio of independent directors to the number of directors. The number of directors measures the board size.

## 4. Empirical Results and Analysis

### 4.1. Descriptive Statistics

Table 2 reports the descriptive statistics of the main variables. The primary variables’ standard deviations were within the normal range, according to the statistical results of the complete sample description, which showed that the variables were less affected by extreme values. The average value of corporate environmental responsibility was 45.30, the maximum value was 90.87, and the minimum value was 8.54, which shows that each enterprise had significant differences in the performance of corporate environmental responsibility. The average value of relational embeddedness was 6.517, the maximum value was 2881, and the minimum value was 0. The average value of network structural embeddedness was 0.802, the maximum value was 68.055, and the minimum value was 0. This shows significant differences in enterprises’ network embeddedness levels in the green innovation network. The green reputation of most businesses was still at a low level, as seen by the average value of 0.415, the maximum value of five, and the minimum value of 0. Other variables were within the normal range. In addition, this paper tested the variables’ variance expansion coefficient (VIF). The maximum value was 5.59, and the minimum value was 1.01, both of which were less than six, indicating no multicollinearity problem.

### 4.2. Correlation Analysis

The correlation coefficients between the variables are reported in Table 3. The findings indicated that relational embeddedness and corporate environmental responsibility had a positive correlation coefficient (β = 0.049), which was substantially positive at the 1% level (*p* < 0.01) and so preliminarily supported Hypothesis 1a. The relationship between structural embeddedness and corporate environmental responsibility had a positive correlation coefficient (β = 0.140), which was also statistically positive at the 1% level (*p* < 0.01), which first supported Hypothesis 1b.

Secondly, the correlation coefficient between green reputation and corporate environmental responsibility was positive (β = 0.804), and it was significantly positive at the level of 1% (*p* < 0.01), indicating that the mediating effect may have existed. In addition, most control variables had significant correlation coefficients with the explained variables, showing that the choice of controls in this study was compelling. Finally, each variable’s correlation coefficient in this table was lower than one, which also showed no significant multicollinearity issues with this study and was consistent with the findings of the VIF in the above analysis.

### 4.3. Basis Analysis Results

Table 4 shows the basis analysis results. Columns (1) and (3) are the regression results of network embeddedness and corporate environmental responsibility without adding control variables. Columns (2) and (4) add control variables. Column (2) shows that the coefficient of relational embeddedness was significantly positive (β = 0.015, *p* < 0.01), indicating that relational embeddedness was positively related to corporate environmental responsibility. Hypothesis 1a was supported. Column (4) shows that the coefficient of structural embeddedness was significantly positive (β = 0.729, *p* < 0.01), indicating that structural embeddedness was positively related to corporate environmental responsibility. Hypothesis 1b was supported.

### 4.4. Mediating Effect Test

Table 5 shows the test results of the mediating effect of green reputation. Column (1) shows that relational embeddedness was directly related to corporate environmental responsibility. The results showed that the coefficient of relational embeddedness was positive and significant at 1%, indicating that green innovation network relational embeddedness can significantly improve corporate environmental responsibility. The relational embeddedness and green reputation coefficients were statistically positive at 1% in column (2). In column (3), relational embeddedness and green reputation were positively related to corporate environmental responsibility at 1%. Existing research indicates that the mediating effect is significant when the coefficient of relational embeddedness in column (1), the coefficient of relational embeddedness in column (2), and the coefficient of green reputation in column (3) are all significantly positive. In addition, the Sobel test and Bootstrap test were further used in this paper. The Bootstrap test had 500 samples, a Z value of 4.405 for the Sobel test, a *p* value of 0.05 or less, and a BC confidence interval after a deviation adjustment of [0.0018053, 0.0222497], excluding 0. Therefore, the research results of this search were robust. To sum up, relational embeddedness had a significant indirect effect on corporate environmental responsibility through green reputation. Hypothesis 2a was supported.

Column (4) shows the direct effect of structural embeddedness on corporate environmental responsibility. The results showed that the coefficient of structural embeddedness was positive and significant at 1%, indicating that structural embeddedness can significantly improve corporate environmental responsibility. In column (5), structural embeddedness positively related to green reputation at 1%. In column (6), structural embeddedness and green reputation were positively related to corporate environmental responsibility at 1%. According to previous studies, the mediating effect is substantial when the coefficients of structural embeddedness in column (4), column (5), and column (6) are significantly positive, as well as when the coefficient of green reputation in column (4) and column (5) are significantly positive. The Sobel test and the Bootstrap test were also utilized in this paper. The Bootstrap test had 500 samples, a Z value of 7.8 for the Sobel test, a *p* value of 0 or less than 0.05, and a BC confidence interval after a deviation adjustment of [0.2927432, 0.6830738], excluding 0. Therefore, the research results of this research were robust. To sum up, structural embeddedness had a significant indirect effect on corporate environmental responsibility through green reputation. Hypothesis 2b was supported.

### 4.5. Moderating Effect Test

Table 6 shows the test results of the moderating effect of ethical leadership. The results in column (1) show that the interaction item of relational embeddedness and ethical leadership (NR × Eth) had a significant positive impact on green reputation. Therefore, ethical leadership positively moderated the relationship between relational embeddedness and green reputation. Thus, Hypothesis 3a was supported. The results of column (2) show that the interaction item of structural embeddedness and ethical leadership (NS × Eth) had a significant positive impact on green reputation. Therefore, ethical leadership positively moderated the relationship between structural embeddedness and green reputation. Therefore, Hypothesis 3b was supported.

### 4.6. Robustness Test

1. Endogenous test

Although the benchmark regression results showed that the higher the degree of network embeddedness was, the higher the corporate environmental responsibility was, endogenous issues still might have an impact on this outcome. In order to verify the robustness of the conclusions of this research, the following methods were adopted to control the potential endogenous problem. The benchmark regression controlled the year effect and industry effect by employing a fixed effect model. As a result, the endogenetic issues that missing factors could bring about were somewhat under control. However, there may have still been interfered with by the reverse causal problems. This means that firms with high environmental responsibility may have tended to embed in the green innovation network. Therefore, the lag-independent variable regression method used in this research could ensure causality to a certain extent. The results are shown in Table 7. The results showed that the regression results of the lagged independent variables were still robust. It can be seen that the impact of the two dimensions of network embeddedness on corporate environmental responsibility was gradually waning by comparing the coefficients of network embeddedness in various lagged years.

2. Sensitivity test of key variables

In order to further verify the robustness of the research, we used substitution independent variable and dependent variable measurement methods. First of all, Table 8 displays the outcomes of calculating the green innovation network embeddedness index using the annual time unit. In columns (1) and (4), the two dimensions of network embeddedness had significant positive effects on corporate environmental responsibility. Hypothesis 1a and 1b were supported again. In columns (2) and (5), the two dimensions of network embeddedness had a significant positive impact on green reputation. In columns (3) and (6), both the two dimensions of network embeddedness and green reputation had significant positive effects on corporate environmental responsibility, indicating that green reputation played a mediating role between network embeddedness and corporate environmental responsibility. Hypothesis 2a and 2b were supported again.

Secondly, referring to the existing research, we used Bloomberg ESG Disclosure Scores as the alternative variable of corporate environmental responsibility. The Bloomberg data came from transparent data of Bloomberg and the third party, which could objectively reflect the ESG situation of the enterprise. The results are shown in Table 9. In columns (1) and (4), the two dimensions of network embeddedness had significant positive effects on corporate environmental responsibility. Hypothesis 1a and 1b were supported again. In columns (2) and (5), the two dimensions of network embeddedness had a significant positive impact on the green reputation. In columns (3) and (6), both the two dimensions of network embeddedness and green reputation had significantly favorable effects on corporate environmental responsibility, indicating that green reputation played a mediating role between network embeddedness and corporate environmental responsibility. Hypothesis 2a and 2b were supported again.

### 4.7. Further Analysis

1. Political connection

First, as a crucial relational resource [82], resource dependence theory states that the corporation must take more precautions to minimize the danger of losing resources the more valuable the resources are to the organization [83]. Compared with companies with lower political affiliations, companies with higher political affiliations can benefit from the convenience of financing and relevant policy support. As these companies gain a lot from political connections, government departments have higher expectations for companies with political connections [84]. Therefore, high-level political-related businesses will interact with government agencies more frequently than low-level businesses, and they will be more driven to take on social responsibility as networks for green innovation become more embedded. If the company’s chairman or general manager was selected by the government, is a current or former government official, serves as a deputy in the National People’s Congress, or is a member of the CPPCC, the political connection was measured as 1; otherwise, it was measured as 0.

This paper distinguishes enterprise samples according to the political association level of enterprises and explored the impact of green innovation network embeddedness on corporate environmental responsibility under different political association levels. The regression results are shown in Table 10. The findings of columns (1) and (3) demonstrate that the sample of high-level political associations had a more significant coefficient of relational embeddedness. The results of columns (2) and (4) demonstrated that the sample of high-level political associations had a more significant coefficient of structural embeddedness. The regression results showed that enterprises with high-level political connections embedded in the green innovation network paid more attention to undertaking corporate environmental responsibility.

2. Financing constraints

The financing constraints faced by enterprises will have a significant impact on their behavior. High financial limitations force an organization to focus on its core business, lowering its investment in corporate social responsibility. When the enterprise faces low financing constraints, it can have sufficient funds so that it will pay more attention to long-term development. At this time, the enterprise has the ability to invest funds to undertake social responsibility. Therefore, this paper predicts that for enterprises with low financing constraints, green innovation network embeddedness may have a more significant effect on promoting corporate environmental responsibility.

Therefore, the sample was split into two groups based on the annual industry average of the company size: high and low financing limitations. This article used company size as an indicator to measure the level of corporate finance restraints. The regression results are shown in Table 11. Only structural embeddedness was significantly positively correlated with corporate environmental responsibility in the high financing constraint samples, and its coefficient was lower than that of the low financing constraint samples. In the low financing constraint samples, network embeddedness was significantly positively correlated with corporate environmental responsibility. This shows that in the lower sample of financing constraints, enterprises embedded in green innovation networks were more conducive to social responsibility.

3. Ownership

The current statistics showed that since 2012, the level of social responsibility of state-owned enterprises was almost more significant than that of non-state-owned enterprises. For state-owned enterprises, due to their solid political atmosphere, they will promote enterprises to actively assume social responsibility [85]. Non-state-owned businesses primarily engage in corporate environmental responsibility to improve their brand recognition or build political connections to lower the cost of debt financing [86]. This paper argues that different ownership determines different incentive mechanisms, which may lead to different effects on enterprises’ embedded networks.

Therefore, this paper distinguished the samples according to the ownership of enterprises and explored the impact of green innovation network embeddedness on corporate environmental responsibility under different ownership samples. The regression results are shown in Table 12. The results of column (1) and column (3) show that the coefficient of relational embeddedness of non-state-owned enterprises was greater than that of state-owned enterprises. The results of column (2) and column (4) show that the coefficient of structural embeddedness of non-state-owned enterprises was more significant than that of state-owned enterprises, and the coefficient was more significant. The results showed that non-state-owned enterprises paid more attention to the long-term development of enterprises by green innovation network embeddedness to actively assume corporate environmental responsibility.

## 5. Conclusions and Discussion

Chinese businesses must balance their own goals with the broader interests of society while engaging in corporate environmental responsibilities against the backdrop of institutional and economic upheaval. Therefore, how to better undertake environmental responsibility is a common concern for academia and industries. Recently, an increasing number of businesses have incorporated the concept of “green innovation” into their concept of corporate social responsibility and engage in corporate environmental responsibility by incorporating the green innovation network. Therefore, in order to further illuminate the internal workings of the advantages of green innovation, this study developed the research framework of “green innovation network embeddedness green reputation corporate environmental responsibility” and extensively examined the marginal change in the indirect effect of green reputation on the relationship between green innovation network embeddedness and corporate environmental responsibility within the constraints of ethical leadership. We took Y02 and Y04 classifications as essential indicators of green patents and built a green innovation network based on the green patents issued by Chinese A-share listed companies from 2010 to 2020. The result showed that relational and structural embeddedness could significantly promote corporate environmental responsibility. In terms of a mediating mechanism, green innovation network embeddedness aided in enhancing enterprises’ green reputation, and green reputation supported firms’ commitment to corporate environmental responsibility. Further analysis showed that ethical leadership positively regulated the intermediary effect of green reputation between green innovation network embeddedness and corporate environmental responsibility. In addition, embeddedness in green innovation networks in fostering corporate environmental responsibility was especially apparent in enterprises with high-level political ties, no financial constraints, and nonstate ownership.

### 5.1. Implications for Theory

First, the study of network embeddedness theory in green innovation was broadened by proposing the idea of a “green innovation network” and employing extensive sample data for empirical testing. The existing research mainly focuses on the impact of network embeddedness on green innovation [87,88]. Few scholars combined “green innovation” with “innovation network” in concept, focusing on the concept of “green innovation network”. In addition, even though the benefits of network embeddedness in enhancing business performance was extensively covered in the literature [16,17,20], few studies used the method of extensive sample empirical tests to give empirical evidence for this aim; instead, they still relied primarily on theoretical analysis and questionnaire surveys. Based on the sample data of Chinese A-share listed companies engaged in green innovation, we built a green innovation network based on the green patents between enterprises. We empirically tested the economic consequences of the embeddedness of green innovation networks. Therefore, based on the perspective of green innovation, this study provides new research ideas for innovation networks. Through empirical research, it tested how green innovation network embeddedness improves corporate environmental responsibility, expanding the research of network embeddedness theory in green innovation.

Second, from the perspective of corporate environmental responsibility, this study expanded the research on the mechanism of green innovation network embeddedness on corporate performance. Previous studies mainly focused on the economic performance of enterprises. First, financial performance includes indicators such as net profit rate, return on equity, and return on assets [16,17]. Second, innovation performance indicators include the number of new items on the market, the productivity of new products in sales, the effectiveness of innovation, and the number of patents [18,20]. There is still a lack of research on the impact of network embeddedness on corporate social performance. This study found that green innovation network embeddedness can significantly improve corporate environmental responsibility performance. Therefore, we broadened both the theoretical application of network embeddedness and the research on the impact of network embeddedness on enterprise performance.

Third, this study put forward the research framework of “green innovation network embeddedness, green reputation, corporate environmental responsibility”. It compensated for the current dearth of research on the impact of green innovation network embeddedness on corporate environmental responsibility and its mechanism by naturally merging resource-based theory, network embeddedness theory, and corporate environmental responsibility. Previous research on network embeddedness mainly explained the formation reason of network embeddedness from the perspective of “resource” acquisition. However, it did not reveal the mechanism of network embeddedness for enterprises to acquire “green resources”. This study offers a fresh viewpoint on how corporate environmental responsibility and embeddedness in green innovation networks interact internally. It also thoroughly explains the transmission function of green reputation in these two domains.

Finally, this study further explored the boundary mechanism of ethical leadership in the green innovation network embeddedness to affect corporate environmental responsibility through green reputation. The existing research on ethical leadership primarily focuses on how managers’ honesty, altruism, trustworthiness, and other qualities affect the creativity of specific employees and teams [68,69] and how to enhance corporate culture [89]. It pays less attention to the research on ethical leadership in network embeddedness. This study included ethical leadership in the logical framework of “green innovation network embeddedness, green reputation, corporate environmental responsibility”. The study confirmed Fu’s [32] view that in the sustainable development strategy, the individual differences of leaders can affect the extent to which enterprises make beneficial activities by demonstrating that the intermediary effect of green reputation between embeddedness in green innovation networks and environmental responsibility will change as a result of leader characteristics. Furthermore, this paper also expands the application of leadership behavior theory in network embeddedness research.

### 5.2. Practical Implications

Firstly, enterprises should attach great importance to the network embedding strategy of green innovation for corporate environmental responsibility, actively integrate the concept of green development into network relationship embedding and network structure embedding, and constantly improve the degree of network relationship embedding and network embedding. In addition, given that the process of green innovation embedding is also a process in which enterprises’ green innovation practice transitions from “light green” to “dark green”, enterprises should reasonably weigh resource input according to their strategic orientation, optimize the strategic combination of green innovation network embedding according to the connotation difference between relational embedding and structural embedding, and adopt scientific and reasonable strategic decisions on green innovation networks to ensure the effective development of green innovation network activities. Especially, the enterprises with low-level political ties, high financing restrictions, and state ownership should focus on environmental responsibility in order to better achieve the sustainable development of the company.

Secondly, enterprises should fully realize the scientific nature of integrating green innovation network embedding, green reputation, ethical leadership, and corporate environmental responsibility into the enterprise performance management framework; adopt scientific and reasonable strategic decisions; pay attention to the construction of external cooperation network; establish and improve communication channels with the government, customers, and other stakeholders; ensure the effective implementation of green innovation network activities; actively establish a good green image; and strive to clarify the requirements and expectations of social participants for corporate environmental responsibility. In addition, enterprises should adopt the strategy of embedding a green innovation network with highly integrated ethics and environmental regulations, create a good cultural environment for corporate environmental responsibility, consciously cultivate and promote ethical leaders with environmental and humanistic perspectives, and encourage these leaders to spread such views to their employees.

Thirdly, the development of enterprises cannot be separated from the support of the external institutional environment. Government departments should fully realize that they should provide necessary environmental incentive policies according to the development needs of enterprises. While green innovation is environmentally sound, it requires significant upfront investment and takes time to transform business models. Therefore, in addition to environmental regulations, the government should also guide entrepreneurs to actively participate in various environmental protection associations and green management training through government–enterprise cooperation, help enterprises establish and strengthen their sense of social responsibility, formulate various incentive policies to constantly encourage enterprises to invest more resources in long-term green innovation network activities, and create a favorable policy environment for the sustainable development of a green innovation network. Thus, enterprises are encouraged to take the initiative to fulfill their environmental and social responsibilities. In addition, the government needs to realize that the effective implementation of environmental mechanism policies such as green subsidies cannot be separated from the cultivation and improvement of the environmental ethics of business leaders. Therefore, the government should incorporate business leaders’ ethical and moral evaluation into the allocation process of incentive policies such as green subsidies.

### 5.3. Limitations and Suggestions for Future Research

There are still some limitations in this study that need to be discussed in the future. Firstly, this study only explored the impact of solid ties and centrality on corporate environmental responsibility. On this basis, subsequent studies can introduce network characteristic variables such as relationship quality, network size, and network density to improve the scope of this study. Secondly, this study only discussed the social performance of network embeddedness from the perspective of corporate environmental responsibility. Subsequent studies can explore how network embeddedness affects corporate social performance, such as green governance, service quality, and employee responsibility. These factors also play an essential role in the long-term development of enterprises. Thirdly, in practice, this study can also include some other significant contingency factors that will affect the boundary conditions of green innovation network embeddedness and corporate environmental responsibility, such as green certification, media attention, and government regulation, to further explore the boundary conditions for enterprises to enhance the green reputation and corporate environmental responsibility. This study only examined the boundary conditions of network embeddedness from ethical leaders. Finally, the samples of this paper were listed companies engaged in green innovation. Different industries have different incentives to participate in the green innovation network. For example, one of the reasons for the rapid development of the new energy vehicle industry in China is government subsidies, which will lead to specific issues related to the green innovation network embeddedness. Therefore, subsequent research can focus on a specific industry.

## Figures and Tables

**Table 1:** Variable definition and measurement.

Variable Type	Variable Name	Variable Symbol	Measurement	Data Sources
Dependent variable	Corporate environmental responsibility	CER	Environmental responsibility scores in the social responsibility report evaluation system of Hexun	www.hexun.com
Independent variable	Relational embeddedness	NR	Number of patent applications jointly filed by enterprises and partners in the network	IncoPat Patent Database
	Structural embeddedness	NS	For degree centrality, the calculation formula is C (n_i_) = d (n_i_)/n − 1	IncoPat Patent Database
Mediating variable	Green reputation	GR	Environmental management system certification score	www.hexun.com
Moderating variable	Ethical leadership	Eth	Sum of scores for humanistic care orientation and environmentally sustainable development orientation	www.hexun.com
Control variable	Firm age	Age	Length of time from the establishment date to the observation period	WIND Database
	Capital structure	Lev	The ratio of total liabilities to total assets	
	Ownership	Soe	For state-owned enterprises, the assigned value is 1, and for non-state-owned enterprises, the assigned value is 0	
	Operating profit ratio	Profit	The ratio of operating profit to total operating income	
	Enterprise growth	Grow	The year-on-year growth rate of operating income	
	The proportion of independent directors	Indep	The ratio of the number of independent directors to the number of directors	
	Company value	CV	It is measured by the ratio of the market value of the owner’s equity and liabilities to the company’s total assets.	
	Cash holdings	Cash	The ratio between the sum of monetary capital and short-term investment and the book value of total assets minus the difference between short-term investment of monetary capital	
	Board size	Board	Number of Directors	
	Year	Year	Year dummy variable	
	Industry	Ind	Industry dummy variable	

**Table 2:** The statistical description of variables.

Variable	N	Mean	Sd	Min	P50	Max
CER	7639	45.30	16.71	8.54	31.56	90.87
NR	7639	6.517	58.62	0	2	2881
NS	7639	0.802	2.804	0	0.725	68.055
GR	7639	0.415	1.204	0	0	5
Eth	7639	5.752	15.28	0	1	52
Age	7639	16.83	5.679	3	17	33
Soe	7639	0.402	0.490	0	0	1
Lev	7639	0.451	0.197	0.074	0.449	0.896
Grow	7639	15.35	22.20	−20.08	12.47	71.03
Indep	7639	0.369	0.068	0	0.333	0.571
Board	7639	8.379	2.526	0	9	15
Cash	7639	0.991	1.504	0.005	0.517	12.00
CV	7639	1	0.004	0.882	1	1.072
Profit	7639	0.093	0.129	−0.474	0.083	0.484

**Table 3:** Pearson correlation analysis.

	CER	NR	NS	GR	Eth	Age	Soe	Lev	Grow	Indep	Board	Cash	CV	Profit
CER	1													
NR	0.049 ***	1												
NS	0.140 ***	0.608 ***	1											
GR	0.804 ***	0.056 ***	0.126 ***	1										
Eth	0.865 ***	0.038 ***	0.121 ***	0.920 ***	1									
Age	−0.055 ***	0.025 **	0.016	−0.053 ***	−0.068 ***	1								
Soe	0.127 ***	0.030 ***	0.071 ***	0.186 ***	0.200 ***	0.101 ***	1							
Lev	−0.051 ***	0.026 **	0.044 ***	0.124 ***	0.118 ***	0.068 ***	0.355 ***	1						
Grow	0.084 ***	−0.018	−0.020 *	−0.025 **	−0.012	−0.176 ***	−0.142 ***	−0.052 ***	1					
Indep	0.015	0.001	0.012	0.014	0.016	0.026 **	0.036 ***	0.020 *	−0.040 ***	1				
Board	0.172 ***	0.060 ***	0.090 ***	0.156 ***	0.165 ***	0.178 ***	0.269 ***	0.083 ***	−0.085 ***	−0.112 ***	1			
Cash	0.061 ***	−0.027 **	−0.050 ***	−0.044 ***	−0.035 ***	−0.147 ***	−0.174 ***	−0.575 ***	0.015	−0.022 *	−0.019 *	1		
CV	−0.021 *	−0.054 ***	−0.070 ***	−0.015	−0.013	0.001	−0.007	−0.037 ***	−0.005	0.015	−0.044 ***	0.017	1	
Profit	0.306 ***	−0.007	−0.011	−0.025 **	−0.012	−0.101 ***	−0.153 ***	−0.419 ***	0.268 ***	−0.063 ***	−0.071 ***	0.311 ***	0.005	1

Note: * *p* < 0.1, ** *p* < 0.05, *** *p* < 0.01.

**Table 4:** The results of the primary effect test.

	(1)	(2)	(3)	(4)
	CER	CER	CER	CER
NR	0.015 ***	0.012 ***		
	(4.88)	(4.28)		
NS			0.729 ***	0.605 ***
			(11.36)	(10.23)
Age		0.252 ***		0.245 ***
		(7.32)		(7.14)
Soe		3.611 ***		3.556 ***
		(9.15)		(9.07)
Lev		5.710 ***		5.689 ***
		(4.66)		(4.68)
Grow		−0.007		−0.006
		(−0.87)		(−0.68)
Indep		15.392 ***		15.073 ***
		(6.15)		(6.06)
Board		0.878 ***		0.862 ***
		(9.19)		(9.08)
CV		−56.177		−37.137
		(−1.21)		(−0.81)
Cash		−0.286 **		−0.236 *
		(−2.01)		(−1.67)
Profit		30.059 ***		29.902 ***
		(12.73)		(12.74)
cons	36.712 ***	61.666	34.208 ***	40.949
	(28.18)	(1.33)	(25.99)	(0.89)
Year	YES	YES	YES	YES
Ind	YES	YES	YES	YES
N	7639	7639	7639	7639
R^2^	0.127	0.292	0.139	0.301

Note: *t* statistics in parentheses; * *p* < 0.1, ** *p* < 0.05, *** *p* < 0.01.

**Table 5:** The results of mediating effect test.

	(1)	(2)	(3)	(4)	(5)	(6)
	CER	GR	CER	CER	GR	CER
NR	0.012 ***	0.001 ***	0.005 ***			
	(4.28)	(4.41)	(2.67)			
NS				0.605 ***	0.037 ***	0.221 ***
				(10.23)	(7.82)	(6.67)
GR			10.714 ***			10.674 ***
			(127.73)			(127.21)
Age	0.252 ***	0.010 ***	0.140 ***	0.245 ***	0.010 ***	0.138 ***
	(7.32)	(3.86)	(7.29)	(7.14)	(3.72)	(7.19)
Soe	3.611 ***	0.255 ***	0.762 ***	3.556 ***	0.251 ***	0.749 ***
	(9.15)	(8.19)	(3.44)	(9.07)	(8.08)	(3.39)
Lev	5.710 ***	0.702 ***	−1.580 **	5.689 ***	0.704 ***	−1.553 **
	(4.66)	(7.29)	(−2.30)	(4.68)	(7.33)	(−2.27)
Grow	−0.007	−0.001	0.004	−0.006	−0.001	0.004
	(−0.87)	(−1.64)	(0.79)	(−0.68)	(−1.53)	(0.90)
Indep	15.392 ***	0.499 **	9.902 ***	15.073 ***	0.480 **	9.802 ***
	(6.15)	(2.54)	(7.09)	(6.06)	(2.45)	(7.04)
Board	0.878 ***	0.042 ***	0.396 ***	0.862 ***	0.042 ***	0.393 ***
	(9.19)	(5.63)	(7.41)	(9.08)	(5.60)	(7.38)
CV	−56.177	−0.539	−45.434 *	−37.137	0.246	−39.104
	(−1.21)	(−0.15)	(−1.76)	(−0.81)	(0.07)	(−1.52)
Cash	−0.286 **	−0.010	−0.183 **	−0.236 *	−0.007	−0.165 **
	(−2.01)	(−0.88)	(−2.31)	(−1.67)	(−0.62)	(−2.09)
Profit	30.059 ***	0.603 ***	23.857 ***	29.902 ***	0.595 ***	23.830 ***
	(12.73)	(3.26)	(18.15)	(12.74)	(3.22)	(18.17)
Constant	61.666	0.366	53.245 **	40.949	−0.518	46.271 *
	(1.33)	(0.10)	(2.07)	(0.89)	(−0.14)	(1.80)
Year	YES	YES	YES	YES	YES	YES
Ind	YES	YES	YES	YES	YES	YES
N	7639	7639	7639	7639	7639	7639
R^2^	0.292	0.186	0.784	0.301	0.190	0.785
Sobel-Z	4.405			7.8		
Goodman-P	0			0		
Intermediary effect	71%			63%		
Bootstrap (BC)	[0.0018053, 0.0222497]			[0.2927432, 0.6830738]		

Note: *t* statistics in parentheses; * *p* < 0.1, ** *p* < 0.05, *** *p* < 0.01.

**Table 6:** The results of moderating effect test.

	(1)	(2)
	GR	GR
NR	0.004 ***	
	(2.61)	
NS		0.237 ***
		(7.49)
Eth	0.918 ***	0.913 ***
	(155.11)	(150.18)
Interact	0.001 **	1.737 *
	(2.38)	(1.70)
Age	0.080 ***	0.078 ***
	(5.46)	(5.36)
Soe	−0.080	−0.084
	(−0.47)	(−0.50)
Lev	−3.951 ***	−3.913 ***
	(−7.75)	(−7.72)
Grow	0.018 ***	0.019 ***
	(5.07)	(5.29)
Indep	5.785 ***	5.655 ***
	(5.51)	(5.42)
Board	0.356 ***	0.351 ***
	(8.77)	(8.70)
CV	−39.417 **	−32.634 *
	(−2.01)	(−1.67)
Cash	−0.355 ***	−0.333 ***
	(−6.06)	(−5.73)
Profit	40.880 ***	40.699 ***
	(61.39)	(61.47)
Constant	49.549 **	42.101 **
	(2.52)	(2.15)
Year	YES	YES
Ind	YES	YES
N	7639	7639
R2	0.834	0.836

Note: *t* statistics in parentheses; * *p* < 0.1, ** *p* < 0.05, *** *p* < 0.01.

**Table 7:** The results of the endogenous test.

	(1)	(2)	(3)	(4)	(5)	(6)
	CER	CER	CER	CER	CER	CER
L.NR	0.023 ***					
	(4.81)					
L2.NR		0.017 ***				
		(2.96)				
L3.NR			0.010			
			(1.48)			
L.NS				0.672 ***		
				(9.55)		
L2.NS					0.460 ***	
					(6.57)	
L3.NS						0.259 ***
						(3.44)
Age	0.229 ***	0.220 ***	0.174 ***	0.221 ***	0.213 ***	0.170 ***
	(6.42)	(6.04)	(4.71)	(6.23)	(5.88)	(4.60)
Soe	3.184 ***	2.683 ***	2.066 ***	3.112 ***	2.628 ***	2.036 ***
	(7.82)	(6.51)	(5.01)	(7.68)	(6.40)	(4.94)
Lev	5.699 ***	4.187 ***	3.553 ***	5.619 ***	4.119 ***	3.504 ***
	(4.51)	(3.27)	(2.76)	(4.47)	(3.23)	(2.72)
Grow	−0.009	−0.012	−0.013	−0.007	−0.010	−0.012
	(−1.00)	(−1.31)	(−1.45)	(−0.83)	(−1.16)	(−1.30)
Indep	16.139 ***	15.571 ***	11.777 ***	15.557 ***	15.068 ***	11.332 ***
	(5.74)	(4.82)	(3.28)	(5.56)	(4.68)	(3.16)
Board	0.791 ***	0.707 ***	0.526 ***	0.791 ***	0.709 ***	0.526 ***
	(7.63)	(6.26)	(4.28)	(7.68)	(6.30)	(4.29)
CV	−92.941 *	−127.424 **	−111.302 **	−72.298	−112.995 **	−105.388 *
	(−1.78)	(−2.23)	(−2.03)	(−1.39)	(−1.98)	(−1.92)
Cash	−0.228	−0.246	−0.224	−0.170	−0.202	−0.199
	(−1.46)	(−1.45)	(−1.22)	(−1.09)	(−1.19)	(−1.08)
Profit	26.321 ***	23.301 ***	19.984 ***	26.220 ***	23.241 ***	20.006 ***
	(10.68)	(9.33)	(7.94)	(10.69)	(9.33)	(7.96)
_ cons	101.993 *	140.722 **	127.027 **	79.679	125.208 **	120.611 **
	(1.95)	(2.46)	(2.31)	(1.53)	(2.19)	(2.20)
Year	YES	YES	YES	YES	YES	YES
Ind	YES	YES	YES	YES	YES	YES
N	6586	5879	5099	6586	5879	5099
R^2^	0.305	0.311	0.328	0.312	0.315	0.329

Note: *t* statistics in parentheses; * *p* < 0.1, ** *p* < 0.05, *** *p* < 0.01.

**Table 8:** Sensitivity test of the independent variable.

	(1)	(2)	(3)	(4)	(5)	(6)
	CER	GR	CER	CER	GR	CER
NR1	0.044 ***	0.003 ***	0.017 ***			
	(5.07)	(4.59)	(3.14)			
NS1				0.480 ***	0.0305 ***	0.158 ***
				(9.45)	(7.61)	(5.52)
			10.711 ***			10.684 ***
			(127.71)			(127.24)
Age	0.253 ***	0.010 ***	0.140 ***	0.247 ***	0.010 ***	0.139 ***
	(7.33)	(3.88)	(7.30)	(7.20)	(3.75)	(7.23)
Soe	3.619 ***	0.255 ***	0.765 ***	3.590 ***	0.252 ***	0.759 ***
	(9.18)	(8.20)	(3.45)	(9.14)	(8.14)	(3.43)
Lev	5.564 ***	0.692 ***	−1.632 **	5.539 ***	0.694 ***	−1.607 **
	(4.55)	(7.18)	(−2.38)	(4.55)	(7.22)	(−2.35)
Grow	−0.008	−0.001 *	0.004	−0.007	−0.001	0.004
	(−0.89)	(−1.68)	(0.77)	(−0.80)	(−1.61)	(0.82)
Indep	15.455 ***	0.504 **	9.926 ***	15.345 ***	0.495 **	9.894 ***
	(6.18)	(2.56)	(7.11)	(6.16)	(2.52)	(7.10)
Board	0.877 ***	0.043 ***	0.397 ***	0.855 ***	0.041 ***	0.392 ***
	(9.19)	(5.67)	(7.43)	(9.00)	(5.53)	(7.34)
CV	−54.545	−0.589	−45.165 *	−40.836	0.108	−41.335
	(−1.18)	(−0.16)	(−1.75)	(−0.89)	(0.03)	(−1.61)
Cash	−0.290 **	−0.010	−0.185 **	−0.266 *	−0.009	−0.177 **
	(−2.04)	(−0.91)	(−2.33)	(−1.88)	(−0.78)	(−2.24)
Roa	61.773 ***	0.309	57.883 ***	60.984 ***	0.263	57.640 ***
	(11.93)	(0.76)	(20.06)	(11.83)	(0.65)	(20.00)
Profit	30.054 ***	0.603 ***	23.857 ***	30.044 ***	0.603 ***	23.877 ***
	(12.74)	(3.26)	(18.15)	(12.79)	(3.27)	(18.19)
Constant	60.105	0.424	53.004 **	45.382	−0.339	48.826 *
	(1.30)	(0.12)	(2.06)	(0.98)	(−0.09)	(1.90)
Year	YES	YES	YES	YES	YES	YES
Ind	YES	YES	YES	YES	YES	YES
N	7639	7639	7639	7639	7639	7639
R^2^	0.293	0.186	0.784	0.299	0.190	0.785

Note: *t* statistics in parentheses; * *p* < 0.1, ** *p* < 0.05, *** *p* < 0.01.

**Table 9:** Sensitivity test of the dependent variable.

	(1)	(2)	(3)	(4)	(5)	(6)
	Social	GR	Social	Social	GR	Social
NR	0.017 ***	0.001 ***	0.016 ***			
	(6.41)	(4.42)	(5.19)			
NS				0.711 ***	0.0367 ***	0.597 ***
				(11.59)	(7.84)	(10.07)
GR			3.544 ***			3.474 ***
			(24.69)			(24.31)
Age	0.264 ***	0.010 ***	0.230 ***	0.251 ***	0.010 ***	0.220 ***
	(7.02)	(3.85)	(6.37)	(6.73)	(3.71)	(6.14)
Soe	5.549 ***	0.254 ***	4.632 ***	5.474 ***	0.250 ***	4.579 ***
	(13.71)	(8.17)	(11.86)	(13.62)	(8.06)	(11.80)
Lev	15.963 ***	0.688 ***	13.305 ***	15.974 ***	0.691 ***	13.391 ***
	(12.80)	(7.27)	(11.07)	(12.91)	(7.32)	(11.21)
Grow	−0.026 ***	−0.001	−0.022 **	−0.024 ***	−0.001	−0.021 **
	(−2.95)	(−1.55)	(−2.55)	(−2.80)	(−1.44)	(−2.44)
Indep	25.591 ***	0.485 **	23.568 ***	25.070 ***	0.467 **	23.155 ***
	(8.91)	(2.48)	(8.57)	(8.80)	(2.39)	(8.47)
Board	1.438 ***	0.042 ***	1.280 ***	1.429 ***	0.042 ***	1.279 ***
	(13.81)	(5.59)	(12.82)	(13.84)	(5.57)	(12.90)
CV	−262.666 ***	−0.531	−238.797 ***	−232.988 ***	0.254	−217.196 ***
	(−4.51)	(−0.15)	(−4.27)	(−4.03)	(0.07)	(−3.91)
Cash	0.115	−0.011	0.180	0.183	−0.008	0.236
	(0.74)	(−1.04)	(1.21)	(1.19)	(−0.77)	(1.59)
Profit	19.857 ***	0.713 ***	16.890 ***	19.320 ***	0.694 ***	16.519 ***
	(12.46)	(5.81)	(11.03)	(12.21)	(5.67)	(10.85)
Constant	238.727 ***	0.381	215.262 ***	207.639 ***	−0.505	192.361 ***
	(4.10)	(0.10)	(3.85)	(3.59)	(−0.14)	(3.46)
Year	YES	YES	YES	YES	YES	YES
Ind	YES	YES	YES	YES	YES	YES
N	7639	7639	7639	7639	7639	7639
R^2^	0.225	0.186	0.297	0.237	0.190	0.306

Note: *t* statistics in parentheses; ** *p* < 0.05, *** *p* < 0.01.

**Table 10:** The impact of political linkages.

	Low-Level Political Connection		High-Level Political Connection	
	(1)	(2)	(3)	(4)
	CER	CER	CER	CER
NR	0.016 ***		0.056 ***	
	(3.66)		(2.67)	
NS		0.483 ***		0.956 ***
		(6.67)		(4.26)
Age	0.239 ***	0.235 ***	0.118	0.112
	(4.59)	(4.52)	(0.99)	(0.95)
Soe	3.809 ***	3.772 ***	5.073 ***	4.769 ***
	(6.83)	(6.79)	(3.57)	(3.37)
Lev	7.954 ***	7.983 ***	24.249 ***	23.407 ***
	(4.62)	(4.66)	(6.14)	(5.94)
Grow	−0.010	−0.009	−0.070 ***	−0.066 ***
	(−0.86)	(−0.78)	(−2.98)	(−2.82)
Indep	14.180 ***	14.030 ***	26.136 **	27.344 **
	(3.02)	(3.00)	(2.31)	(2.43)
Board	0.791 ***	0.781 ***	1.243 ***	1.344 ***
	(5.05)	(5.01)	(3.32)	(3.59)
CV	−293.28 ***	−257.07 **	124.161	135.346
	(−2.93)	(−2.57)	(0.96)	(1.05)
Cash	−0.478 **	−0.431 **	0.284	0.316
	(−2.45)	(−2.21)	(0.75)	(0.84)
Profit	25.699 ***	25.625 ***	13.114	15.543 *
	(7.51)	(7.52)	(1.47)	(1.75)
Constant	300.531 ***	262.894 ***	−142.992	−156.898
	(2.99)	(2.63)	(−1.10)	(−1.21)
Year	YES	YES	YES	YES
Ind	YES	YES	YES	YES
N	4370	4370	3269	3269
R^2^	0.284	0.289	0.374	0.381

Note: *t* statistics in parentheses; * *p* < 0.1, ** *p* < 0.05, *** *p* < 0.01.

**Table 11:** The impact of financing constraints.

	High Financing Constraints		Low Financing Constraints	
	(1)	(2)	(3)	(4)
	CER	CER	CER	CER
NR	0.001		0.009 ***	
	(0.13)		(2.92)	
NS		0.434 **		0.383 ***
		(2.06)		(5.61)
Age	0.063 *	0.064 *	0.272 ***	0.263 ***
	(1.68)	(1.71)	(5.18)	(5.02)
Soe	1.472 ***	1.476 ***	2.566 ***	2.566 ***
	(3.17)	(3.18)	(4.64)	(4.66)
Lev	−3.496 ***	−3.412 **	−0.569	−0.177
	(−2.61)	(−2.54)	(−0.29)	(−0.09)
Grow	−0.005	−0.005	0.005	0.007
	(−0.64)	(−0.56)	(0.43)	(0.51)
Indep	3.612	3.533	13.917 ***	13.844 ***
	(1.48)	(1.44)	(3.45)	(3.45)
Board	0.413 ***	0.424 ***	0.735 ***	0.727 ***
	(3.83)	(3.94)	(5.36)	(5.32)
CV	41.724	38.592	−26.157	−15.816
	(0.50)	(0.46)	(−0.47)	(−0.29)
Cash	0.008	0.020	−0.692 **	−0.623 **
	(0.07)	(0.16)	(−2.48)	(−2.24)
Profit	28.073 ***	27.920 ***	22.303 ***	22.422 ***
	(11.81)	(11.74)	(6.13)	(6.18)
Constant	−24.810	−22.331	45.996	33.485
	(−0.30)	(−0.27)	(0.83)	(0.61)
Year	Year	Year	Year	Year
Ind	Year	Year	Year	Year
N	3522	3522	4117	4117
R^2^	0.284	0.285	0.357	0.360

Note: *t* statistics in parentheses; * *p* < 0.1, ** *p* < 0.05, *** *p* < 0.01.

**Table 12:** The impact of ownership.

	Non-State-Owned Enterprises		State-Owned Enterprise	
	(1)	(2)	(3)	(4)
	CER	CER	CER	CER
NR	0.012 **		0.011 ***	
	(2.28)		(2.99)	
NS		0.699 ***		0.486 ***
		(7.31)		(6.11)
Age	0.353 ***	0.324 ***	0.140 **	0.155 **
	(9.08)	(8.33)	(2.20)	(2.43)
Lev	8.669 ***	8.231 ***	2.653	3.030
	(5.99)	(5.72)	(1.21)	(1.38)
Grow	−0.014	−0.013	0.009	0.011
	(−1.46)	(−1.41)	(0.56)	(0.71)
Indep	12.663 ***	12.464 ***	20.219 ***	19.967 ***
	(4.53)	(4.48)	(4.43)	(4.39)
Board	0.731 ***	0.765 ***	0.997 ***	0.949 ***
	(6.20)	(6.52)	(6.58)	(6.28)
CV	−65.777	−47.417	−44.683	−29.119
	(−0.99)	(−0.72)	(−0.68)	(−0.45)
Cash	0.197	0.205	−0.703 *	−0.553
	(1.40)	(1.47)	(−1.68)	(−1.32)
Profit	23.794 ***	23.714 ***	31.196 ***	31.206 ***
	(8.69)	(8.71)	(7.53)	(7.57)
Constant	75.290	56.764	55.742	38.585
	(1.13)	(0.86)	(0.85)	(0.59)
Year	YES	YES	YES	YES
Ind	YES	YES	YES	YES
N	4301	4301	3308	3308
R^2^	0.240	0.248	0.363	0.369

Note: *t* statistics in parentheses; * *p* < 0.1, ** *p* < 0.05, *** *p* < 0.01.

## Data Availability

IncoPat Patent Database; www.hexun.com; WIND Datebase.
